# Genes and Athletic Performance: The 2023 Update

**DOI:** 10.3390/genes14061235

**Published:** 2023-06-08

**Authors:** Ekaterina A. Semenova, Elliott C. R. Hall, Ildus I. Ahmetov

**Affiliations:** 1 Department of Molecular Biology and Genetics, Federal Research and Clinical Center of Physical-Chemical Medicine of Federal Medical Biological Agency, 119435 Moscow, Russia; 2 Research Institute of Physical Culture and Sport, Volga Region State University of Physical Culture, Sport and Tourism, 420138 Kazan, Russia; 3 Faculty of Health Sciences and Sport, University of Stirling, Stirling FK9 4UA, UK; 4 Laboratory of Genetics of Aging and Longevity, Kazan State Medical University, 420012 Kazan, Russia; 5 Sports Genetics Laboratory, St Petersburg Research Institute of Physical Culture, 191040 St. Petersburg, Russia; 6 Department of Physical Education, Plekhanov Russian University of Economics, 115093 Moscow, Russia; 7 Research Institute for Sport and Exercise Sciences, Liverpool John Moores University, Liverpool L3 5AF, UK

**Keywords:** sports, genetics, genotype, polymorphism, genomics, physical performance, athletes, GWAS, WGS, WES

## Abstract

Phenotypes of athletic performance and exercise capacity are complex traits influenced by both genetic and environmental factors. This update on the panel of genetic markers (DNA polymorphisms) associated with athlete status summarises recent advances in sports genomics research, including findings from candidate gene and genome-wide association (GWAS) studies, meta-analyses, and findings involving larger-scale initiatives such as the UK Biobank. As of the end of May 2023, a total of 251 DNA polymorphisms have been associated with athlete status, of which 128 genetic markers were positively associated with athlete status in at least two studies (41 endurance-related, 45 power-related, and 42 strength-related). The most promising genetic markers include the *AMPD1* rs17602729 C, *CDKN1A* rs236448 A, *HFE* rs1799945 G, *MYBPC3* rs1052373 G, *NFIA-AS2* rs1572312 C, *PPARA* rs4253778 G, and *PPARGC1A* rs8192678 G alleles for endurance; *ACTN3* rs1815739 C, *AMPD1* rs17602729 C, *CDKN1A* rs236448 C, *CPNE5* rs3213537 G, *GALNTL6* rs558129 T, *IGF2* rs680 G, *IGSF3* rs699785 A, *NOS3* rs2070744 T, and *TRHR* rs7832552 T alleles for power; and *ACTN3* rs1815739 C, *AR* ≥21 CAG repeats, *LRPPRC* rs10186876 A, *MMS22L* rs9320823 T, *PHACTR1* rs6905419 C, and *PPARG* rs1801282 G alleles for strength. It should be appreciated, however, that elite performance still cannot be predicted well using only genetic testing.

## 1. Introduction

Athletic success is influenced by many genetically determined factors, including transcriptomic, biochemical, histological, anthropometric, physiological, and psychological traits, as well as general health status [1,2,3,4,5,6,7,8]. On average, 66% of the variance in athlete status can be explained by genetic factors [9]. The remaining variance is due to environmental factors, such as deliberate practice, nutrition, ergogenic aids, birthplace, the availability of medical and social support, and even luck (e.g., birthdate) [6,10,11,12].

Starting in the late 1990s, research began to identify DNA polymorphisms associated with predisposition to certain types of sports and exercise-related phenotypes, with initial focus on variants of the *ACE*, *ACTN3*, *AMPD1*, *PPARA*, *PPARD*, and *PPARGC1A* genes [13,14,15,16,17,18,19,20,21,22,23]. Initially, most research was conducted using the candidate gene approach [24,25,26,27,28,29], which limited progress in the discovery of new genetic markers associated with exercise- and sport-related phenotypes [30]. In addition to the fact that this approach studies only a single genetic variant in isolation, most candidate gene studies in the field of sports genomics are limited by sample size. This is a potential source of type I error (false positive findings), underpinning why replication of positive associations in independent cohorts is essential. Conversely, the genome-wide association approach is considered the most efficient study design thus far in identifying genetic markers associated with sport-related characteristics. Indeed, the application of this approach has enabled the discovery of hundreds of single nucleotide polymorphisms (SNPs) directly or indirectly associated with exercise and sport, such as height (12,111 SNPs) [31], appendicular lean mass (1059 SNPs) [32], testosterone levels (855 SNPs) [33], handgrip strength (170 SNPs) [34,35,36], sarcopenia (78 SNPs) [37], and brisk walking (70 SNPs) [38].

It is important to consider that the discovery of 12,111 independent genome-wide (*p* < 5 × 10^−8^) significant SNPs associated with height (which in combination account for 40% of phenotypic variance) required the study of 5.4 million individuals [31]. Accordingly, such findings indicate that each DNA locus is likely to explain only a very small proportion of the reported phenotypic variance (~0.0033%). This also suggests that very large samples of athletes and non-athletic controls are needed to detect a substantial number of genetic markers associated with athlete status, with some suggesting those markers be used as part of talent identification strategies. To date, five genome-wide association (GWAS) studies [39,40,41,42,43], one whole-genome sequencing (WGS) study [44], and one exome-wide association (EWAS) study [45] involving athletic cohorts have been conducted. However, due to their limited sample sizes, these investigations and subsequent replication studies have resulted in the identification of only 13 genetic markers. Six markers were associated with endurance athlete status (*CDKN1A* rs236448 A, *GALNTL6* rs558129 C, *NFIA-AS2* rs1572312 C, *MYBPC3* rs1052373 G, *SIRT1* rs41299232 G, and *TRPM2* rs1785440 G alleles) [39,40,42,43,45,46], five with sprinter/strength athlete status (*AGRN* rs4074992 C, *CDKN1A* rs236448 C, *CPNE5* rs3213537 G, *NUP210* rs2280084 A, and *GALNTL6* rs558129 T alleles) [41,45,47,48], and two with reaction time in wrestlers and athlete status in combat/team sports (*APC* rs518013 A and *LRRN3* rs80054135 T alleles, respectively) [44].

**Figure 1:**
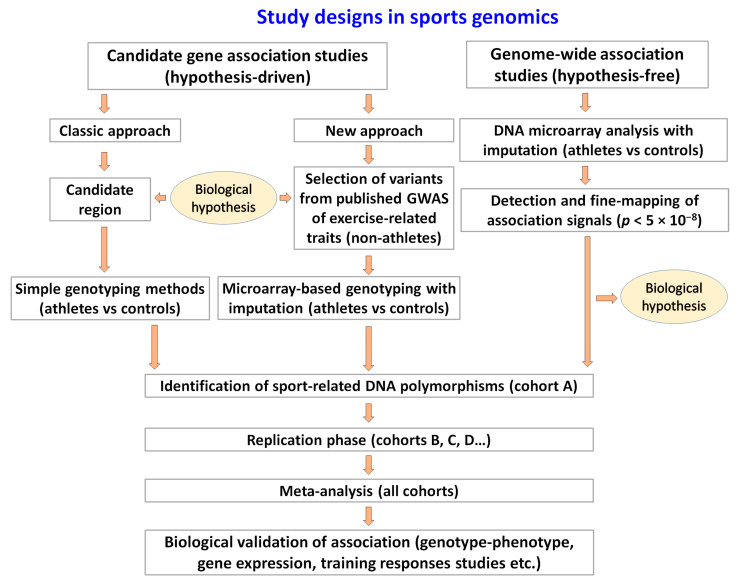
Case-control study designs in sports genomics. In this approach, allelic frequencies are compared between athletes and controls (e.g., endurance athletes vs. untrained subjects or endurance vs. power athletes). A case-control study may be the first step followed by a genotype–phenotype study (e.g., identification of VO_2max_ or weightlifting performance-increasing genotypes among athletes). In some cases, studies begin with a genotype–phenotype approach, and the findings are subsequently validated by a case-control study.

To overcome the issue of small samples of elite athletes, the use of genome-wide significant (*p* < 5 × 10^−8^) markers of exercise-related phenotypes (correlated with sport-related traits) discovered in large cohorts of untrained subjects (for example, UK Biobank, FinnGen, BioBank Japan, etc.) was proposed [49,50]. This approach may significantly decrease the risk of obtaining false-positive results in the discovery of markers associated with athlete status, which is recognized as a key limitation of sports genomics studies conducted on limited sample sizes. Accordingly, the first stage of such an investigation is to list candidate genetic markers previously identified in GWAS of exercise-related phenotypes in non-athletic populations. For example, this may include SNPs associated with phenotypes of handgrip strength, brisk walking, physical activity, lean mass, forced vital capacity, haemoglobin, or levels of hormones such as testosterone and IGF1. At the second stage, microarray analysis (with imputation; covering > 10 million SNPs) is carried out in a group of athletes to test the hypothesis that favourable alleles of exercise-related phenotypes are over-represented in those athletes compared to non-athletic controls (Figure 1). This approach may equally be applied to determine whether favourable alleles could be associated with direct measures of athletic capability, such as weightlifting performance, running times, or scores recorded during competitive events. In this case, the significance threshold for the second stage is set at *p* < 0.05 (to reduce the likelihood of potentially important findings being overlooked).

A recent example of such an approach being implemented is the study by Guilherme et al. [49], which investigated two correlated phenotypes: brisk walking pace (using UK Biobank participants) and sprint athlete status (using elite Russian sprinters). Brisk walkers perform more physical activity, are taller, have reduced adiposity and demonstrate greater physical performance and strength versus slower walkers, with such traits also recorded more commonly in sprinters than other athletes of other disciplines. Therefore, it was hypothesized that the alleles associated with high-speed walking (discovered in untrained subjects) would also be over-represented in elite sprinters. Accordingly, 70 genetic markers of brisk walking were identified from the literature [38], of which 15 SNPs had a significantly different allele frequency when comparing sprinters with non-athletic controls [49]. The same innovative approach later identified 23 SNPs associated with strength athlete status [51] based on genome-wide significant markers for handgrip strength in a non-athletic population using the UK Biobank [34,35]. Furthermore, using a panel of 822 testosterone-related SNPs from the UK Biobank study [33], five DNA-polymorphisms associated with muscle fibre size and weightlifting performance were identified [50].

Another approach that has proven effective in addressing the possibility of false positive results in sports genomics literature is to perform replication studies in two or more independent athletic cohorts (even with small or moderate sample sizes), followed by a meta-analysis to quantify the overall effect of a polymorphism on athlete status and/or a sport- and exercise-related trait [43,52,53,54,55,56,57,58,59,60,61,62,63,64,65]. However, in some cases, replication is not possible due to the exclusivity of a polymorphism to specific populations based on their geographic ancestry. For example, the rs671 G/A polymorphism of the aldehyde dehydrogenase 2 (*ALDH2*) gene was associated with strength in athletes and non-athletes from the Japanese population [66,67,68]. Interestingly, the unfavourable (associated with reduced strength) rs671 A allele is not present in Europeans or South Asians (frequency 0%), but common in Chinese, Japanese, and Vietnamese populations (15–25%). This demonstrates a notable challenge seeking to replicate genomic findings in larger samples, as increasing the study sample must also consider the geographic ancestry of participants. This also highlights the possibility that the genetic determinants of some sport- and exercise-related phenotypes are restricted to certain populations, demonstrating that increasing sample size is not as straightforward as simply recruiting participants from multiple countries and/or continents.

As well as the phenotypes of athlete status or competitive performance, several recent studies have investigated a broader range of traits which may relate directly or indirectly to athletic capability. These include flexibility, coordination, cardiorespiratory fitness, spatial ability, stress resilience, mental toughness, fat loss efficiency, and cardiovascular and metabolic responses to training, amongst others [69,70,71,72,73,74,75,76,77,78,79,80,81,82,83,84]. For example, combat athletes are more likely than untrained subjects to have the warrior (*COMT* rs4680 GG) genotype [85], whilst chess players demonstrate an increased frequency of an allele linked to improved memory and spatial ability (*KIBRA* rs17070145 T) [86]. Such discoveries demonstrate the broadening nature of sports genomics in recent times, with focus expanding from the traditional domain of investigating what makes elite performers different from the general population into other domains, such as sports nutrigenetics [87,88,89,90,91,92,93,94,95,96,97,98,99,100] and areas of sports medicine, such as genomic variants associated with soft-tissue injuries and sports-related concussion [101,102,103,104,105,106,107,108,109,110,111,112,113,114].

Technological advancement has lowered the cost of conducting genomic studies, increasing accessibility to researchers who wish to investigate the genetic underpinnings of sport and exercise phenotypes. Consequently, sports genomics is a dynamic and continually developing field, making it important to regularly appraise the contribution of recent advances to the field. Therefore, the aim of the current review was to summarise recent progress in understanding the genetic determinants of athlete status, and to detail novel DNA polymorphisms that may underpin differences between individuals in their athletic potential.

**Figure 2:**
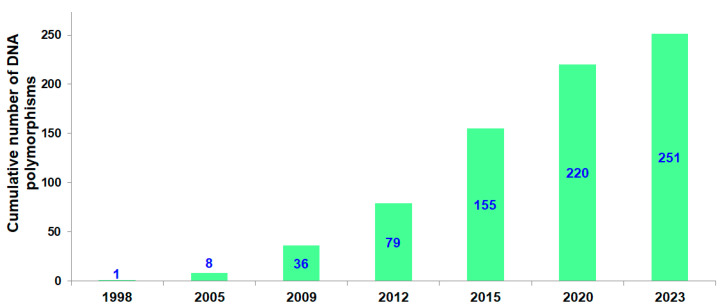
Sports-related genetic markers discovered between 1998 and 2023.

At the time of writing (end of May 2023), the total number of DNA polymorphisms associated with athletic performance since the first discovery in 1998 is 251 (Figure 2). Our search for sports genomics publications was based on journals indexed in major databases (i.e., PubMed etc.) using specific key words (e.g., athletes + polymorphism/genotype etc.). However, not all articles were included in the current review due to language limitations (articles written in languages other than English must contain at least abstracts in English). In addition, papers with very small cohort (less than 25 in athletes/controls), or articles with combined groups of athletes (for example, endurance + power without separation) were not included. Abstracts of conference proceedings were not considered. In recognition of the fact that many studies in the field of sports genomics report associations based on the investigation of small sample sizes, we stipulated that only markers where statistically significant associations have been reported in at least two studies (two case-control studies and/or one case-control plus one functional study; including those presented in one article) would be included in the present review.

According to these criteria, 128 markers could be associated with athlete status (41 endurance-related, 45 power-related, and 42 strength-related) from the original 251 identified in our literature search. The most promising genetic markers (i.e., most replicated and had fewer negative or controversial findings) include *AMPD1* rs17602729 C, *CDKN1A* rs236448 A, *HFE* rs1799945 G, *MYBPC3* rs1052373 G, *NFIA-AS2* rs1572312 C, *PPARA* rs4253778 G, and *PPARGC1A* rs8192678 G alleles for endurance; *ACTN3* rs1815739 C, *AMPD1* rs17602729 C, *CDKN1A* rs236448 C, *CPNE5* rs3213537 G, *GALNTL6* rs558129 T, *IGF2* rs680 G, *IGSF3* rs699785 A, *NOS3* rs2070744 T, and *TRHR* rs7832552 T alleles for power; and *ACTN3* rs1815739 C, *AR* ≥ 21 CAG repeats, *LRPPRC* rs10186876 A, *MMS22L* rs9320823 T, *PHACTR1* rs6905419 C, and *PPARG* rs1801282 G alleles for strength. This update on the panel of genetic markers associated with athlete status covers advances in research reported in the past two years (previous online version was published in 2021 [115]). The current review also lists all known markers associated with endurance, power, or strength athlete status/performance. This article does not aim to review genetic markers associated with team (game) and combat sports, markers for which are well described elsewhere [26,61,116,117].

## 2. Gene Variants for Endurance Athlete Status

An individual’s endurance capacity is determined by many factors, including their muscle fibre typology, haemoglobin mass, mitochondrial biogenesis, maximal cardiac output, and maximal rate of oxygen consumption (VO_2max_), among others [118,119,120,121,122,123,124]. Indeed, there is evidence that these intermediate phenotypes have a substantial genetic influence, with literature indicating that genetic factors account for up to 70% of the variability in endurance-related traits [125]. Usually, genetic markers associated with endurance athlete status are determined by comparing allelic frequencies between endurance athletes (e.g., biathletes, road cyclists etc.) and controls.

To support the observed findings from endurance-related case-control studies, researchers subsequently perform functional, lab-based studies to determine the relationship between genotypes and physiological measures. Examples of measurements used to complement genomic studies include (but are not limited to) VO_2max_, forced expiratory volume in one second (FEV1), proportion of slow-twitch muscle fibres, recovery speed, long-distance running performance, running economy, lactate threshold, erythropoietin and haemoglobin levels, number of erythrocytes, capillary density, mitochondrial density, fat metabolism, and fatigue resistance.

Our literature search revealed that at least 41 of the 114 reported markers could be associated with endurance athlete status based on our criteria (Table 1). The most promising genetic markers for endurance athlete status include *AMPD1* rs17602729 C, *CDKN1A* rs236448 A, *HFE* rs1799945 G, *MYBPC3* rs1052373 G, *NFIA-AS2* rs1572312 C, *PPARA* rs4253778 G, and *PPARGC1A* rs8192678 G alleles. In contrast, the other 73 markers (endurance alleles) have not passed our strict criteria: *ACOXL* rs13027870 G, *ADRA2A* 6.7kb, *ADRA2A* rs1800544 G, *ADRB1* rs1801252 G, *AGT* rs699 A, *BDKRB2* rs1799722 T, *CAMK1D* rs11257754 A, *CHRNB3* rs4950 G, *CLSTN2* rs2194938 A, *CNDP2* rs6566810 A, *COL5A1* rs71746744 AGGG, *COL6A1* rs35796750 T, *CPQ* rs6468527 A, *CYP2D6* rs3892097 G, *DMT1* 258 bp, *EPAS1 (HIF2A)* rs1867785 G, *EPAS1 (HIF2A)* rs11689011 T, *GABPB1* rs8031031 T, *GALM* rs3821023 A, *GNB3* rs5443 T, *GRM3* rs724225 G, *GSTT* rs17856199 (+), *IGF1R* rs1464430 A, *IL6* rs1800795 C, *IL15RA* rs2228059 A, *ITPR1* rs1038639 T, *ITPR1* rs2131458 T, *FMNL2* rs12693407 G, *KCNJ11* rs5219 C, *L3MBTL4* rs17483463 T, *MSTN* rs11333758 D, MtDNA loci (G1, HV, L0, M*, m.11215T, m.152C, m.15518T, m.15874G, m.4343G, m.514(CA) ≤ 4, poly(C ≥ 7) stretch at m.568–573, m.16080G, m.5178C, N9, V, unfavourable: B, J2, T, L3*), *NALCN-AS1* rs4772341 A, *NACC2* rs4409473 C, *NATD1* rs732928 G, *NOS3* rs1799983 G, *NOS3* (CA)n 164bp, *NOS3* 27bp 4B, *PPARD* rs2016520 C, *PPARD* rs1053049 T, *PPARGC1A* rs4697425 A, *PPARGC1B* rs11959820 A, *PPP3CA* rs3804358 C, *PPP3CB* rs3763679 C, *SGMS1* rs884880 A, *SLC2A4* rs5418 A, *SOD2* rs4880 C, *SPOCK1* rs1051854 T, *TPK1* rs10275875 T, *TTN* rs10497520 T, Y-chromosome haplogroups (E*, E3*, and K*(xP); unfavourable: E3b1), and *ZNF429* rs1984771 G. Most of these markers have been described in previous reviews [27,126,127] but cannot be included in our current list of endurance-associated markers until they are validated through replication by additional studies.

## 3. Gene Variants for Power Athlete Status

Several characteristics are positively associated with power performance, including circulating levels of testosterone, percentage and cross-sectional area of fast-twitch muscle fibres, muscle mass and strength, body and calcaneus height, muscle fascicle length, and reaction time, among others [3,238,239,240,241,242,243,244]. The heritability of power-related phenotypes has been reported in the literature to range from approximately 49 to 86% in a range of phenotypes, including jumping ability [245,246]. Typically, genetic markers associated with power athlete status are determined by comparing allelic frequencies between power athletes (e.g., 100 m runners, shot putters, arm wrestlers, etc.) and untrained subjects. To support findings from case-control studies, investigators perform genotype–phenotype studies by measuring sprint times, jump performance, muscle fibre size, muscle fibre typology, maximal strength, rate of force development, and circulatory levels of anabolic hormones such as testosterone. Our literature search revealed that at least 45 of the 95 markers reportedly associated with power athlete status met our new criteria (Table 2). The most promising of these genetic markers associated with power athlete status currently include *ACTN3* rs1815739 C, *AMPD1* rs17602729 C, *CDKN1A* rs236448 C, *CPNE5* rs3213537 G, *GALNTL6* rs558129 T, *IGF2* rs680 G, *IGSF3* rs699785 A, *NOS3* rs2070744 T, and *TRHR* rs7832552 T alleles. In contrast, the remaining 50 genetic markers (power alleles) did not meet our strict criteria: *ARHGEF28* rs17664695 G, *CACNG1* rs1799938 A, *CALCR* rs17734766 G, *CLSTN2* rs2194938 C, *CNDP1* rs2887 A, *CNDP1* rs2346061 C, *CNDP2* rs3764509 G, *COTL1* rs7458 T, *CREM* rs1531550 A, *DMD* rs939787 T, *EPAS1* (*HIF2A*) rs1867785 G, *EPAS1* (*HIF2A*) rs11689011 C, *FOCAD* rs17759424 C, *GABRR1* rs282114 A, *GALNT13* rs10196189 G, *GPC5* rs852918 T, *IGF1R* rs1464430 C, *IL1RN* rs2234663 *2, *IP6K3* rs6942022 C, *MCT1* rs1049434 A, *MED4* rs7337521 T, *MPRIP* rs6502557 A, MtDNA loci (favourable: F, m.204C, m.151T, m.15314A, Non-L/U6, unfavourable: m.16278T, m.5601T, m.4833G, m.5108C, m.7600A, m.9377G, m.13563G, m.14200C, m.14569A), *MTR* rs1805087 G, *MTRR* rs1801394 G, *NOS3* rs1799983 G, *NRG1* rs17721043 A, *PPARGC1A* rs8192678 A, *PPARGC1B* rs10060424 C, *RC3H1* rs767053 G, *SLC6A2* rs1805065 C, *SUCLA2* rs10397 A, *TPK1* rs10275875 C, *UCP2* rs660339 C, *VEGFR2* rs1870377 T, *WAPL* rs4934207 C, and *ZNF423* rs11865138 C. The majority of these markers are reported in previous reviews [25,126,127] and should be validated in additional studies before they can meet the criteria to be included in our list of power-associated genetic variants.

## 4. Gene Variants for Strength Athlete Status

Performance in strength-based sports is based on multiple factors. However, the factors considered to contribute substantially to strength phenotypes include skeletal muscle hypertrophy (muscle fibre size), hyperplasia, the predominance of fast-twitch muscle fibres, a greater muscle fascicle pennation angle, improved neurological adaptation, high glycolytic capacity, and increased circulatory testosterone [297]. Importantly, evidence exists that strength athletes exhibit vastly different transcriptomic, biochemical, anthropometric, physiological, and biomechanical characteristics compared to endurance athletes and/or controls [1,4]. These differences can be explained by the presence of both deliberate environmental (training, nutrition, etc.) and genetic factors. Indeed, studies indicate that there is a strong heritability of power- and strength-related traits, where genetic factors account for up to 85% of the variation in maximal isometric, isotonic, and isokinetic strength [246]. In a recent study investigating the genetic component of severe sarcopenia (the age-related decline in skeletal muscle mass, strength, and gait speed) [37], it was found that the alleles associated with higher risk of severe sarcopenia were closely linked to tiredness, alcohol intake, smoking, time spent watching television, and a higher self-reported consumption of salt and processed meat. In contrast, alleles associated with lower risk of severe sarcopenia were positively associated with levels of serum testosterone, IGF1, and 25-hydroxyvitamin D; height; physical activity; as well as indicators of healthier dietary habits (self-reported intake of cereal, cheese, oily fish, protein, water, fruit, and vegetables). Whilst muscle strength phenotypes in the general population may be less pronounced than in strength athletes, the latter may represent an ideal population to identify genomic variants associated with skeletal muscle capacity, potentially aiding the advancement of knowledge surrounding sarcopenia and directing strategies to reduce the negative impact of age-related declines in muscle mass. In general, genetic markers associated with strength athlete status can be determined by comparing allelic frequencies between strength athletes and controls. To support these findings, scientists perform genotype–phenotype studies by measuring handgrip and isokinetic strength, powerlifting/weightlifting performance, as well as evaluating the acute and chronic responses to resistance training.

Previously, 170 DNA polymorphisms were reported to be associated with handgrip strength in three large GWASs [34,35,36]. In a follow-up study involving elite weightlifters and powerlifters, Moreland et al. [51] tested the hypothesis that alleles associated with greater handgrip strength would be over-represented in these athletes compared to controls. Accordingly, they identified 23 DNA polymorphisms that were associated with strength athlete status. Of these SNPs, the *LRPPRC* rs10186876, *MMS22L* rs9320823, and *PHACTR1* rs6905419 polymorphisms were also associated with superior competitive weightlifting performance [298].

Our literature search based on our new inclusion criteria revealed at that least 42 genetic markers could be associated with strength athlete status (Table 3). The most promising genetic markers for strength athlete status include *ACTN3* rs1815739 C, *AR* ≥ 21 CAG repeats, *LRPPRC* rs10186876 A, *MMS22L* rs9320823 T, *PHACTR1* rs6905419 C, and *PPARG* rs1801282 G alleles.

## 5. Conclusions

The current review demonstrates that at least 251 genetic markers are reportedly linked to sport-related traits. However, only 128 (51%) of these markers (41 endurance-related, 45 power-related, and 42 strength-related) have been associated with athlete status in two or more studies. On the other hand, of these 128 genetic markers, the significance of 29 (22.7%) DNA polymorphisms was not replicated in at least one study, raising the possibility that a number of findings may represent false positives. It is important to consider that there may be one of several reasons why the findings of a study may not be replicated by another, including disparity of sample sizes, small sample sizes in one or more of the studies, different study designs, inconsistent classification of sporting groups or types of sport (strength, power, etc.), variability in how researchers or research groups define the term “elite athletes” (some researchers define the term “elite” as performances at the international level, others if the athlete is a prize winner in international competitions), and the ethnicity/geographical ancestry of the cohorts studied, amongst others.

As discussed previously, height remains not only the most studied exercise-related phenotype at the genetic level, but also the most studied human trait, with 12,111 associated SNPs [31]. It is estimated that the final number of height-related SNPs may reach 25,000 (with a minor allele frequency of ≥1%), but the sample size needs to be increased to approximately 100 million individuals of the same ethnicity. These values should be noteworthy and serve as a benchmark for the direction of future research in the field of sports genomics, where the current number of 251 genetic markers must be increased by a considerable magnitude in order to fully comprehend the genomic underpinnings of exercise performance, and thus to be considered as potential predictors of talent in sport. Given that effective talent identification remains a challenging task despite decades of research and strategy [322,323,324], it remains possible that the development of predictive genetic performance tests in future may be able to contribute to the advancement of this field. However, the literature currently available does not support the use of genetic testing for these purposes [325,326,327].

Whilst genomics is the among the most established molecular sub-disciplines of sport and exercise research, sport- and exercise-related DNA polymorphisms do not fully explain the heritability of athlete status. Consequently, other forms of variation, such as rare mutations [328,329] and epigenetic markers (i.e., stable and heritable changes in gene expression) [330], must be considered. Newly emerging high-throughput technologies enable the design of multi-omics approaches integrating various -omics levels (metabolomics, transcriptomics, proteomics, epigenomics, etc.) with the aim of determining how each level contributes to the biological mechanisms underpinning physical performance. For example, transcriptomic analyses have revealed the roles of both genomic and epigenomic mechanisms in modulating the transcription of genes regulated by exercise [2,331,332]. Incorporating multi-omics approaches has the potential to drastically advance the understanding of how the acute response to exercise is regulated, and consequently how chronic adaptations to exercise are mediated in the context of elite performance and/or health and wellbeing. Accordingly, future research, including collaborative multicentre GWASs and whole-genome sequencing of large athlete cohorts with further validation and replication, as well as the use of large purpose-built Biobanks, should focus on identifying genetic and other -omics markers of sport-related phenotypes and their underlying biology.

Our review does have limitations. First, we have not provided information regarding genetic markers associated with team (game) and combat sports, flexibility, coordination, personality, cognitive abilities, muscle fibre composition, skeletal muscle hypertrophy, injuries, and responses to training/supplements. These markers are well described elsewhere [4,24,26,28,37,61,74,79,115,116,117,333,334]. Second, we have not described all studies in detail (ethnicity, specific sporting disciplines, sample size, *p*-values etc.) given word limit. Third, some genetic markers (out of the 128 most significant) were selected based on data obtained in case-control studies only, without confirmation of functional significance (genotype–phenotype studies are therefore warranted).

In conclusion, our literature search revealed at least 251 DNA polymorphisms that could be associated with endurance, power, and strength athlete statuses. Most of these genetic markers have been discovered in studies involving Australian, Brazilian, British, Canadian, Chinese, Croatian, Czech, Ethiopian, Finnish, French, German, Greek, Hungarian, Indian, Iranian, Israeli, Italian, Jamaican, Japanese, Kenyan, Korean, Lithuanian, Polish, Qatari, Russian, Slovenian, South African, Spanish, Taiwanese, Tatar, Tunisian, Turkish, Ukrainian, and US athletes.

## Figures and Tables

**Table 1:** Genetic markers for endurance athlete status.

Gene	Full Name	Locus	Polymorphism	Endurance-Related Allele	References	
					Studies with Positive Results	Studies with Negative or Controversial Results
*ACE*	Angiotensin I converting enzyme	17q23.3	Alu I/D (rs4343 A/G or rs4341 C/G)	I (A or C)	[14,15,16,128,129,130,131,132,133,134,135,136,137,138,139,140,141]	[133,142,143,144,145,146,147,148,149,150,151,152,153]
*ACTN3*	Actinin α 3	11q13.1	rs1815739 C/T	T	[17,154,155,156]	[152,157,158,159,160,161,162,163,164,165,166,167,168,169,170]
*ADRB2*	Adrenoceptor β 2	5q31-q32	rs1042713 G/A	A	[160,171,172]	[173,174]
*ADRB2*	Adrenoceptor β 2	5q31-q32	rs1042714 G/C	C	[153,175]	[173,174]
*ADRB3*	Adrenoceptor β 3	8p11.23	rs4994 A/G	G	[170,173]	
*AGTR2*	Angiotensin II receptor type 2	Xq22-q23	rs11091046 A/C	C	[176]	[177]
*AQP1*	Aquaporin 1	7p14	rs1049305 C/G	C	[178,179,180]	
*AMPD1*	Adenosine monophosphate deaminase 1	1p13	rs17602729 C/T	C	[19,153,181,182,183]	[184]
*BDKRB2*	Bradykinin receptor B2	14q32.1-q32.2	+9/−9 (exon 1)	–9	[185,186]	[153,187,188,189]
*CDKN1A*	Cyclin Dependent Kinase Inhibitor 1A	6p21.2	rs236448 A/C	A	[43]	
*CKM*	Creatine kinase M-type	19q13.32	rs8111989 A/G	A	[190,191,192]	[133,193]
*COL5A1*	Collagen type V α 1 chain	9q34.2-q34.3	rs12722 C/T	T	[194,195]	
*FTO*	FTO α-Ketoglutarate Dependent Dioxygenase	16q12.2	rs9939609 T/A	T	[196,197]	[198]
*GABPB1*	GA binding protein transcription factor subunit β 1	15q21.2	rs12594956 A/C	A	[199,200]	
			rs7181866 A/G	G	[199,201]	[200]
*GALNTL6*	Polypeptide N-acetylgalactosaminyltransferase 6	4q34.1	rs558129 T/C	C	[40]	
*GSTP1*	Glutathione S-transferase Pi 1	11q13.2	rs1695 A/G	G	[202,203]	
*HFE*	Homeostatic iron regulator	6p21.3	rs1799945 C/G	G	[153,204,205,206,207]	
*HIF1A*	Hypoxia inducible factor 1 subunit α	14q23.2	rs11549465 C/T	C	[208,209]	[144,210]
*MCT1*	Monocarboxylate transporter 1	1p12	rs1049434 A/T	T	[60,211,212,213,214]	[215]
MtDNA loci	Mitochondrial DNA	MtDNA	MtDNA haplogroups	H	[161,216]	
				Unfavourable: K	[161,216]	
*MYBPC3*	Myosin Binding Protein C3	11p11.2	rs1052373 A/G	G	[42]	
*NFATC4*	Nuclear factor of activated T cells 4	14q11.2	rs2229309 G/C	G	[144]	
*NFIA-AS2*	NFIA antisense RNA 2	1p31.3	rs1572312 C/A	C	[39,46]	
*NOS3*	Nitric oxide synthase 3	7q36	rs2070744 T/C	T	[153,217,218]	[219]
*PPARA*	Peroxisome proliferator activated receptor α	22q13.31	rs4253778 G/C	G	[20,220,221,222]	
*PPARGC1A*	Peroxisome proliferative activated receptor, γ, coactivator 1 α	4p15.1	rs8192678 G/A	G	[18,20,170,223]	[216,224,225]
*PPARGC1B*	Peroxisome proliferative activated receptor, γ, coactivator 1 β	5q32	rs7732671 G/C	C	[144,226]	
*PPP3R1*	Protein phosphatase 3 regulatory subunit B, α	2p15	Promoter 5I/5D	5I	[144,227]	
*PRDM1*	PR/SET Domain 1	6q21	rs10499043 C/T	T	[228,229]	
*RBFOX1*	RNA binding fox-1 homolog 1	16p13.3	rs7191721 G/A	G	[39]	
*SIRT1*	Sirtuin 1	10q21.3	rs41299232 C/G	G	[45]	
*SPEG*	Striated Muscle Enriched Protein Kinase	2q35	rs7564856 G/A	G	[230]	
*TFAM*	Transcription factor A, mitochondrial	10q21	rs1937 G/C	C	[144,231]	[216]
*TRPM2*	Transient Receptor Potential Cation Channel Subfamily M Member 2	21q22.3	rs1785440 A/G	G	[45]	
*TSHR*	Thyroid stimulating hormone receptor	14q31	rs7144481 T/C	C	[39]	
*UCP2*	Uncoupling protein 2	11q13	rs660339 C/T	T	[130,144,232]	
*UCP3*	Uncoupling Protein 3	11q13	rs1800849 C/T	T	[130,144]	[233]
*VEGFA*	Vascular endothelial growth factor A	6p12	rs2010963 G/C	C	[144,234,235]	
*VEGFR2*	Vascular endothelial growth factor receptor 2	4q11-q12	rs1870377 T/A	A	[236,237]	

**Table 2:** Genetic markers for power athlete status.

Gene	Full Name	Locus	Polymorphism	Power-Related Allele	References	
					Studies with Positive Results	Studies with Negative or Controversial Results
*ACE*	Angiotensin I converting enzyme	17q23.3	Alu I/D (rs4343 A/G or rs4341 C/G)	D (G)	[16,128,145,169,247,248,249,250]	[150,251,252,253,254]
*ACVR1B*	Activin A type IB receptor	12q13.13	rs2854464 A/G	A	[255,256]	[256,257]
*ACTN3*	Actinin α 3	11q13.1	rs1815739 C/T	C	[17,161,162,168,170,250,258,259,260,261,262,263,264]	[159,165,254,265,266]
*ADAM15*	ADAM Metallopeptidase Domain 15	1q21.3	rs11264302 G/A	G	[49]	
*ADRB2*	Adrenoceptor β 2	5q31-q32	rs1042713 G/A	G	[41,174]	
			rs1042714 C/G	G	[41,174]	
*AGRN*	Agrin	1p36.33	rs4074992 C/T	C	[45]	
*AGT*	Angiotensinogen	1q42.2	rs699 T/C	C	[41,267,268]	
*AGTR2*	Angiotensin II receptor type 2	Xq22-q23	rs11091046 A/C	A	[176,177]	[55]
*AKAP6*	A-Kinase Anchoring Protein 6	14q12	rs12883788 C/T	C	[49]	
*AMPD1*	Adenosine monophosphate deaminase 1	1p13	rs17602729 C/T	C	[184,269,270]	
*AUTS2*	Activator of Transcription and Developmental Regulator AUTS2	7q11.22	rs10452738 A/G	A	[49]	
*BDNF*	Brain derived neurotrophic factor	11p14.1	rs10501089 G/A	A	[271]	
*CCT3*	Chaperonin Containing TCP1 Subunit 3	1q22	rs11548200 T/C	T	[49]	
*CDKN1A*	Cyclin Dependent Kinase Inhibitor 1A	6p21.2	rs236448 A/C	C	[43]	
*CKM*	Creatine kinase, M-type	19q13.32	rs8111989 A/G	G	[65,272,273]	[274]
*CNTFR*	Ciliary neurotrophic factor receptor	9p13.3	rs41274853 C/T	T	[275]	
*CPNE5*	Copine V	6p21.2	rs3213537 G/A	G	[41,48]	
*CRTAC1*	Cartilage Acidic Protein 1	10q24.2	rs2439823 A/G	A	[49]	
*CRTC1*	CREB Regulated Transcription Coactivator 1	19p13.11	rs11881338 T/A	A	[49]	
*E2F3*	E2F Transcription Factor 3	6p22.3	rs4134943 C/T	T	[49]	
*FHL2*	Four and a Half LIM Domains 2	2q12.2	rs55680124 C/T	C	[49]	
*GALNTL6*	Polypeptide N-acetylgalactosaminyltransferase like 6	4q34.1	rs558129 C/T	T	[47,276]	
*GDF5*	Growth Differentiation Factor 5	20q11.22	rs143384 A/G	G	[49]	
*HIF1A*	Hypoxia inducible factor 1 α subunit	14q21-q24	rs11549465 C/T	T	[277,278,279]	
*HSD17B14*	Hydroxysteroid 17-β dehydrogenase 14	19q13.33	rs7247312 A/G	G	[41]	
*IGF1*	Insulin like growth factor 1	12q23.2	rs35767 C/T	T	[280,281]	
*IGF2*	Insulin like growth factor 2	11p15.5	rs680 A/G	G	[41,281,282]	
*IGSF3*	Immunoglobulin Superfamily Member 3	1p13.1	rs699785 G/A	A	[49]	
*IL6*	Interleukin 6	7p21	rs1800795 C/G	G	[41,283,284]	
*ILRUN*	Inflammation and Lipid Regulator with UBA-Like and NBR1-Like Domains	6p21.31	rs205262 A/G	A	[49]	
*MTHFR*	Methylenetetrahydrofolate reductase	1p36.3	rs1801131 A/C	C	[285,286]	
*NOS3*	Nitric oxide synthase 3	7q36	rs2070744 T/C	T	[219,279,287]	
*NRXN3*	Neurexin 3	14q24.3-q31.1	rs8011870 G/A	G	[49]	
*NUP210*	Nucleoporin 210	3p25.1	rs2280084 C/A	C	[45]	
*PIEZO1*	Piezo Type Mechanosensitive Ion Channel Component 1	16q24.3	rs572934641 (TCC/-) E756del	D	[288]	
*PPARA*	Peroxisome proliferator activated receptor α	22q13.31	rs4253778 G/C	C	[159,220,289]	
*PPARG*	Peroxisome proliferator activated receptor γ	3p25.2	rs1801282 C/G	G	[279,290,291]	[292]
			rs2920503 C/T	T	[49]	
*SLC39A8*	Solute Carrier Family 39 Member 8	4q24	rs13107325 C/T	C	[49]	
*SOD2*	Superoxide dismutase 2	6q25.3	rs4880 C/T	C	[293]	
*TRHR*	Thyrotropin releasing hormone receptor	8q23.1	rs7832552 C/T	T	[65,294,295]	
*UBR5*	Ubiquitin Protein Ligase E3 Component N-Recognin 5	8q22.3	rs10505025 G/A	A	[296]	
			rs4734621 G/A	A	[296]	
*ZNF568*	Zinc Finger Protein 568	19q13.12	rs1667369 A/C	A	[49]	

**Table 3:** Genetic markers for strength athlete status.

Gene	Full Name	Locus	Polymorphism	Strength-Related Allele	References	
					Studies with Positive Results	Studies with Negative or Controversial Results
*ABHD17C*	Abhydrolase domain containing 17C	15q25.1	rs7165759 G/A	A	[35,51]	
*ACE*	Angiotensin I converting enzyme	17q23.3	Alu I/D (rs4343 A/G or rs4341 C/G)	D (G)	[299,300,301,302,303,304]	[305]
*ACTG1*	Actin γ 1	17q25.3	rs6565586 T/A	A	[34,51]	
*ACTN3*	Actinin α 3	11q13.1	rs1815739 C/T	C	[302,306,307,308]	[51,305,309,310]
*ADCY3*	Adenylate cyclase 3	2p23.3	rs10203386 T/A	T	[35,51]	
*ADPGK*	ADP dependent glucokinase	15q24.1	rs4776614 C/G	C	[35,51]	
*AGT*	Angiotensinogen	1q42.2	rs699 T/C	C	[310,311]	[51]
*ALDH2*	Aldehyde Dehydrogenase 2 Family Member	12q24.12	rs671 G/A	G	[66,67,68]	
*ANGPT2*	Angiopoietin 2	8p23.1	rs890022 G/A	A	[51,312]	
*AR*	Androgen Receptor	Xq12	(CAG)n	≥21	[313,314]	
*ARPP21*	CAMP regulated phosphoprotein 21	3p22.3	rs1513475 T/C	C	[35,51]	
*BCDIN3D*	Bicoid interacting 3 domain containing RNA methyltransferase	12q13.12	rs12367809 C/T	C	[35,51]	
*CKM*	Creatine kinase, M-type	19q13.32	rs8111989 A/G	G	[54,315]	[51,274]
*CNTFR*	Ciliary neurotrophic factor receptor	9p13.3	rs41274853 C/T	T	[275,316]	[51]
*CRTAC1*	Cartilage acidic protein 1	10q24.2	rs563296 G/A	G	[35,51]	
*DHODH*	Dihydroorotate dehydrogenase (Quinone)	16q22.2	rs12599952 G/A	A	[35,51]	
*GALNTL6*	Polypeptide N-acetylgalactosaminyltransferase-like 6	4q34.1	rs558129 C/T	T	[47]	
*GBE1*	1, 4-α-glucan branching enzyme 1	3p12.2	rs9877408 A/G	A	[35,51]	
*GBF1*	Golgi brefeldin A resistant guanine nucleotide exchange factor 1	10q24.32	rs2273555 G/A	A	[34,317]	
*GLIS3*	GLIS Family Zinc Finger 3	9p24.2	rs34706136 T/TG	TG	[50]	
*HIF1A*	Hypoxia inducible factor 1 α	14q21-q24	rs11549465 C/T	T	[278,295,318]	[51]
*IGF1*	Insulin-like growth factor 1	12q23.2	rs35767 C/T	T	[51,280,319]	
*IL6*	Interleukin 6	7p21	rs1800795 C/G	G	[51,283]	
*ITPR1*	Inositol 1, 4, 5-Triphosphate Receptor Type 1	3p26.1	rs901850 G/T	T	[35,51]	
*KIF1B*	Kinesin family member 1B	1p36.22	rs11121542 G/A	G	[35,51]	
*LRPPRC*	Leucine-rich pentatricopeptide repeat cassette	2p21	rs10186876 A/G	A	[34,51,298]	
*MLN*	Motilin	6p21.31	rs12055409 A/G	G	[35,317]	
*MMS22L*	Methyl methanesulfonate-sensitivity protein 22-Like	6q16.1	rs9320823 T/C	T	[35,51,298]	
*MTHFR*	Methylenetetrahydrofolate reductase	1p36.3	rs1801131 A/C	C	[51,286,298]	
*NPIPB6*	Nuclear pore complex interacting protein family member B6	16p12.1	rs2726036 A/C	A	[35,51]	
*PHACTR1*	Phosphate and actin regulator 1	6p24.1	rs6905419 C/T	C	[35,51,298]	
*PLEKHB1*	Pleckstrin homology domain containing B1	11q13.4	rs7128512 A/G	G	[51,312]	
*PPARA*	Peroxisome proliferator activated receptor α	22q13.31	rs4253778 G/C	C	[220,320,321]	[51]
*PPARG*	Peroxisome proliferator activated receptor γ	3p25.2	rs1801282 C/G	G	[51,290,291]	
*PPARGC1A*	Peroxisome proliferative activated receptor, γ, coactivator 1 α	4p15.2	rs8192678 G/A	A	[51,305,308]	
*R3HDM1*	R3H domain containing 1	2q21.3	rs6759321 G/T	T	[35,51]	
*RASGRF1*	Ras protein specific guanine nucleotide Releasing Factor 1	15q25.1	rs1521624 C/A	A	[35,51]	
*RMC1*	Regulator of MON1-CCZ1	18q11.2	rs303760 C/T	C	[35,51]	
*SLC39A8*	Solute carrier family 39 member 8	4q24	rs13135092 A/G	A	[35,51]	
*TFAP2D*	Transcriptional factor AP-2 delta	6p12.3	rs56068671 G/T	T	[35,51]	
*ZKSCAN5*	Zinc finger with KRAB and SCAN domains 5	7q22.1	rs3843540 T/C	C	[35,51]	
*ZNF608*	Zinc finger protein 608	5q23.2	rs4626333 G/A	G	[312,317]	

## Data Availability

Not applicable.
